# Promoting medical competencies through international exchange programs: benefits on communication and effective doctor-patient relationships

**DOI:** 10.1186/1472-6920-14-43

**Published:** 2014-03-04

**Authors:** Fabian Jacobs, Karsten Stegmann, Matthias Siebeck

**Affiliations:** 1 Center for International Health CIH, Department of Surgery, Faculty of Medicine, LMU Munich, Nussbaumstr. 20, 80336 Munich, Germany; 2 Department of Psychology, LMU Munich, Leopoldstrasse 13, 80802 Munich, Germany; 3 Department of Surgery, Faculty of Medicine, LMU Munich, Munich, Germany

**Keywords:** Medical education, Sociocultural learning, Vygotsky, International cooperation, Internationalization on universities, Outcomes of exchange programs, Global health education

## Abstract

**Background:**

Universities are increasingly organizing international exchange programs to meet the requirements of growing globalisation in the field of health care. Analyses based on the programs’ fundamental theoretical background are needed to confirm the learning value for participants. This study investigated the extent of sociocultural learning in an exchange program and how sociocultural learning affects the acquisition of domain-specific competencies.

**Methods:**

Sociocultural learning theories were applied to study the learning effect for German medical students from the LMU Munich, Munich, Germany, of participation in the medical exchange program with Jimma University, Jimma, Ethiopia. First, we performed a qualitative study consisting of interviews with five of the first program participants. The results were used to develop a questionnaire for the subsequent, quantitative study, in which 29 program participants and 23 matched controls performed self-assessments of competencies as defined in the Tuning Project for Health Professionals. The two interrelated studies were combined to answer three different research questions.

**Results:**

The participants rated their competence significantly higher than the control group in the fields of doctor-patient relationships and communication in a medical context. Participant responses in the two interrelated studies supported the link between the findings and the suggested theoretical background.

**Conclusion:**

Overall, we found that the exchange program affected the areas of doctor-patient relationships and effective communication in a medical context. Vygotsky’s sociocultural learning theory contributed to explaining the learning mechanisms of the exchange program.

## Background

### Learning through exchange programs: a socio-cultural learning experience

Recently, research on exchange programs for medical students has become more important because the rapid increase in the number of international exchange programs between universities has resulted in discussions about the impact of such exchange programs on medical training [1,2]. Although there have been demands for a further increase in the number of international exchange programs [3] it remains unclear whether exchange programs achieve their objectives, which include helping students obtain certain qualifications to work in a diverse setting, supporting global health knowledge, and promoting the internationalization of universities [4]. Empirical studies on exchange programs have demonstrated different effects on (job-specific) competencies and basic skills [5].

The assumption that the social environment influences what is learned and how things are learned gives rise to questions about the effect of a specific training on exchange program participants and the theoretical basis for the learning process. Palthe and Thomas, Chang, and Abt complain about a lack of theory-based empirical studies on exchange programs [6,7]. Thus, research on exchange programs requires theory-based models that can predict which processes contribute most to the success of exchange programs. A substantiated theoretical background would allow researchers to determine which exchange programs are more effective and to evaluate the educational content offered in the programs on a theoretical basis. Without such a theoretical background, evaluations are without scientific grounding or implications and medical schools cannot know the impact of exchange programs on their participants.

The current research was based on the hypothesis that the social interactions of participants in an exchange program increase job-specific competencies. Against this background, we examined the effects of sociocultural learning in international exchange programs on job-specific competencies and to what extent these effects can be explained against the background of sociocultural learning theories. Vygotsky’s sociocultural approach changed the perspective of learning from an individualistic to a sociocultural one [8] and thus provides a potential conceptual foundation for exchange programs. It provides the theoretical basis for the assumption that exchange programs should affect not only general soft skills but also job-specific competencies in medicine.

The central construct of Lew Vygotsky’s work about how individuals learn is the zone of proximal development (ZPD) [8]. According to Vygostky’s theoretical approach, a driving force for individual cognitive development is the social interaction between learners with different levels of competence [9]. Initially, Vygotsky developed his ideas to explain the cognitive development of children [10]. However, his ideas stimulated research in nearly all areas of social learning. A central aspect of the theoretical basis for the current study is Damon’s assumption that peer-to-peer learning stimulates the learner to consider the environment critically, which subsequently leads to a critical examination and mental conflict [11]. This conflict is developed by contradictions between the learner’s beliefs and the more capable peer’s explanations. As a result of this process, the individual starts to challenge his or her existing knowledge, to reflect on it and to search for new information. This procedure results in a new learning process [12].

In this context, Mietzel [13] noticed that the thoughts, values, and attitudes of the learner develop through interaction with others not by a passive reception, but rather by internalizing active constructions. Also, Mietzel noted that this mental conflict can become an appropriate catalyst for variations or changes in knowledge [13]. Damon points out that peers of the same age are particularly suited to activating such conflicts [11]. These ideas consider different activators than those mentioned originally by Vygotsky and indicate that it is not sufficient for the interaction partner only to be more capable because a learning process involves much more important factors. Central is a mental conflict that leads people to reflect on their personal beliefs and thoughts. Engeström [14] emphasizes situations of crisis as a positive factor for the sequence of learning.

Gutierrez and Stone [15] point out that conflicts, contradictions, and tensions—as certainly are experienced when members of different cultures are together—can lead to a positive and fruitful dialogue of learning. There is a great chance that these conflicts, if they are discussed and debated, lead to a “zone of good cooperation and good learning”. [15].

Meacham [16] commented on the opportunity found in cultural diversity. He pointed out that cultural diversity often has been viewed as the presence of a deficit in the one who is different, but that, in light of the sociocultural theory, diversity actually offers a great learning opportunity [16]. The term “culture” addresses another important aspect: The social environment in which someone is learning is compared with the environment in which someone is acting.

On the basis of Vygotsky’s theoretical framework and the considerations presented above, the main assumption for this research was the idea that participants in an exchange program only gain new knowledge from interacting with capable peers if there is a conflict caused by differences between the participants and their peers. This learning process can be supported by diverse learning cultures.

This led us to the following three research questions (RQ):

RQ1: Which medical skills can be improved by participating in an international exchange program?

RQ2: To what extent does participating in an exchange program affect the self-assessment of medical skills?

RQ3: Which learning processes mediate the effects of exchange programs with regard to self assessment of medical skills?

## Methods

We conducted two different studies to answer the research questions. We decided to use a qualitative interview to investigate RQ1, because of its explorative nature. We then applied a quasi-experimental design to investigate RQ2. However, the two research questions were highly interrelated (i.e. the results of RQ1 influenced which medical skills were tested in RQ2, and the results of the qualitative analysis reported in RQ1 might have validated the quantitative differences found in RQ2). For this reason, we decided to describe the methods of the two studies together and not separately. To answer RQ3, we combined the results of both studies.

### Participants and design

We conducted the research within the context of the Jimma University (JU, Jimma, Ethiopia) – LMU Munich (LMU, Munich, Germany) Link for Medical Education (JU-LMU Link). The priorities of the JU-LMU Link are the exchange of staff and undergraduate students and faculty development through academic teacher training courses. So far, more than one hundred people have participated in the exchange program. The duration of the visits in Jimma were between six and twelve weeks. All participating students were in the clinical part of their undergraduate training. Within the JU-LMU Link German students have the possibility to participate in the community based training program (CBTP) and thereafter in a clinical rotation in a self-chosen clinical field. During the internship they were only in an observing position. During the CBTP a German-Ethiopian group of students work together in a rural area near Jimma. CBTP gives German medical students the chance to participate in an educational program that is not offered in the German educational system, and to experience the living conditions in rural Africa.

In our *interview study*, we interviewed five exchange program participants who were among the first participants (one woman and four men; age range 26–32 years) from the LMU. The interviewees had participated in the exchange program about eight years before the interviews were conducted. We assumed that any effects we found would be long term, because of the long interval between their stay in Ethiopia and the interviews. The qualitative interviews were a pilot measure to assess possible influences of the program from a long-term perspective. All participants were contacted first by email and then telephone and all agreed to participate.

In our *quasi-experimental study*, with an “observational via survey” character we compared people who had participated in the exchange program (n = 29) with a control group consisting of people who had applied for the exchange program but had not been selected because of capacity limits (n = 23). To recruit participants for the study, we contacted all former participants and applicants for the exchange program by email. The email contained a link to an online survey and additional information, e.g. duration and content of the survey, information about the whole survey, and information regarding the anonymity of all data. Because the number of applicants exceeded the number of available places on the exchange program candidates were selected on the basis of their personal ability to stay in a low-income country. Candidates were not selected on the basis of their medical competencies or grades. The participants in the two groups did not differ regarding age, sex, field of study, or previous international experience. A total of 21 men and 30 women participated; one person did not specify if they were a man or woman. The mean age of the exchange program participants was M = 26.9 (SD 3.73); the mean age of the control group, M = 24.93 (SD 4.28).

### Assessing competencies of physicians in medicine

A critical issue in research on exchange programs is the valid and reliable measurement of competencies. To define the areas of knowledge and skills that comprise “competence of physicians in medicine,” we decided to use the outcomes of the Tuning Project. The Tuning Project is an “initiative […] to develop learning outcomes/competencies for degree programmes in Europe and to promote harmonization in the Higher Education Sector” [17]. The project has prepared competence catalogues for different occupational fields, including medicine. The competence catalogue for medicine consists of three different levels of outcomes: The first section consists of 29 generic outcomes for Higher Education degrees, derived from previous phases of the “parent” Tuning Project; the second section consists of 12 discipline-specific Level 1 outcomes, which together encompass the competencies required for medical graduates; and the third section includes a series of discipline-specific Level 2 outcomes (74 in total) for each Level 1 outcome.

### Data sources

We performed the qualitative *interview study* in April to June 2009. Each interview took about 45 to 65 minutes. We conducted two interviews face-to-face and three by telephone. The interviews were theory based and guided by a manual. Before conducting the five interviews, a test interview was conducted with three test persons to evaluated whether participants understood the questions in the guideline manual, whether the order of the questions was optimal, and how long the interview lasted. At the beginning of each interview, one of the investigators (FJ) informed each participant about the anonymity of all data and about the aim of the study.

The manual consisted of 31 open questions, intervening short explanations, and a small quantitative survey, which we based on the Level 1 Tuning competencies questionnaire. The interview guide can be found in the appendix (see Additional file 1).

The *interview study* was important for the development of the quantitative questionnaire to be used in the subsequent study. On the basis of the interviewees’ open answers, we revised the questionnaire items with regard to the Level 1 Tuning competencies. We analyzed the audiotaped interviews by applying a qualitative content analysis according to Mayring [18] and divided the material into units. We conducted the interviews in German and recorded, transcribed and analyzed all the responses in German. The findings have been translated into English for the current research report.

To measure the competencies we administered an online questionnaire. On the basis of the initial interview study four (out of twelve) Tuning skills were assessed. These 4 Tuning skills were chosen because interviewees in the initial *interview study* had indicated that they had improved these skills as a result of the exchange program. In addition, we included questions about four “Outcomes for Medical Professionalism” from the Tuning Project. These Outcomes were selected out of the responses of the interviews.

The four Tuning skills were as follows: “Apply ethical and legal principles in medical practice;” “Assess psychological and social aspects of a patient's illness;” “Communicate effectively in a medical context;” and “Carry out a consultation with a patient.” Since the participants in the interview study had difficulties with these questions, particularly with identifying whether or not they had increased their professional competencies (see Additional file 2) in these areas, we rephrased the statements about these four skills for the online questionnaire.

The four Outcomes for Medical Professionalism were as follows: “Professional attributes” (e. g., interpersonal skills and ethical commitmment), “Professional working” (e. g., problem solving skills and leadership), “The doctor as expert” (e. g., research skills and teaching skills), and “The global doctor” (e. g., second language and general knowledge).

We asked participants to self-assess their current skills on a scale ranging numerically from 1 to 5 and verbally from “strongly disagree” to “strongly agree.” For example, competence in “Communicate effectively in a medical context” was assessed by the following statements: “I can talk appropriately with patients from other cultures,” “I can communicate purposefully in a medical context,” “I have had a lot of exchanges with other professionals about best practices in medical practice,” “My ability to communicate with colleagues is excellent,” “I can have a good conversation with someone who needs an interpreter”. The whole online questionnaire is provided in the appendix and each rephrased statement is visible there (see Additional file 3). The reliability of all scales was sufficiently high (details can be found in Table 1). Means were computed across the different items for each skill/outcome to serve as score for the self-assessed skill/outcome.

**Table 1:** Reliability of the dependent variables

**Dependent variable**	**Number of items**	**Cronbach’s α**
**Tuning skills**		
Apply ethical and legal principles in medical practice	6	α = .69
Assess psychological and social aspects of a patient’s illness	5	α = .77
Lead into effectively in a medical context	2	α = .62
Carry out a consultation with a patient	5	α = .77
**Outcomes for medical professionalism**		
Professional attributes	7	α = .71
Professional working	9	α = .76
The doctor as expert	5	α = .67
The global doctor	4	α = .78

The first part of the questionnaire had to be completed only by participants who had participated in the exchange program. The respective questions were meant to assess the extent of their perceived sociocultural learning during the exchange. To examine RQ2, i.e. “To what extent does participating in an exchange program affect the self assessment of medical skills?,” we applied t-tests.

We investigated RQ 3 (“Which learning processes mediate the effects of exchange programs with regard to the self-assessment of medical skills?”) on the basis of the results of the qualitative and quantitative surveys. The analyses for this research question were of a descriptive nature.

We used the open excess program F4 to transcribe the qualitative interview. All statistical analyses were performed with SPSS 18.

## Results

The qualitative *interview study* provided data that allowed us to answer RQ1 and to develop an interview guideline for the subsequent, quasi-experimental *quantitative study*. Analysis of the data from the *quantitative study* were used to answer RQ2. Finally, data obtained from both studies were combined to explore RQ3.

### RQ 1: which medical skills can be improved by participating in an international exchange program?

The five qualitative interviews indicated the difficulty in determining effects by using the pure Tuning Project Level 1 outcomes (see Additional file 2 for a summary of the results). Asking the interviewees directly about the specific Tuning outcomes identified only minor implications of the exchange program. However, when we considered the interviewees’ responses to the open questions, we were able to attribute a variety of statements to the specific Tuning outcomes. One of the most important areas was the doctor-patient relationship, which all five intereviewees mentioned explicitly. Examples of responses are “*In Africa, the doctor hardly talks to the patient*” (A) #14:33–2# (authors’ translation), and “*You can learn for your own work, how much importance you should still place on a good relationship between the doctor and patient*” (B) #00:13:20–1# (authors’ translation). All interviewees reported that their respective experiences made them more aware of the important role of the doctor-patient relationship in Germany and how important it is for health care in general. For example, interviewee C remarked: “… *that the appreciation of physicians is different in our country, ehm, that’s definitely the case, and, as I already said several times, that it is related to the way doctors deal with patients; then, I think I learned a lot or it confirmed my thoughts also about the attitude to work and responsibility, that some things we have are also very important* “(C) #00:01:59-4# (authors’ translation). The interviewees commented also on the ethical side of the doctor-patient relationship, for example: “*Attitude in front of patients toward ethical aspects; viewing patients as people and not just as a disease*” (E) #00:52:17-8# (authors’ translation).

### RQ 2: to what extent does participating in an exchange program affect the self-assessment of medical skills?

A similar picture emerged in the quantitative online questionnaire, i.e. most respondents reported gains in the area of the doctor-patient relationship.

Table 2 shows the descriptive data of the quantitative survey. The mean rating for competencies in all four Level 1 learning outcomes was higher in those who had participated in the JU-LMU Link exchange program than in the control group.

**Table 2:** Mean and standard deviation of the dependent variables

		**Non-participants (n = 23)**	**Participants (n = 29)**	***p***	**Cohen’s *d***
**Tuning skills**					
Communicate effectively in a medical context	*M*	3.15	3.67	.033	.63
	*SD*	.935	.735		
Apply ethical and legal principles in medical practice	*M*	4.11	4.23	.324	.27
	*SD*	.470	.424		
Assess psychological and social aspects of a patien’s illness	*M*	3.93	4.05	.200	.26
	*SD*	.517	.420		
Carry out a consultation with a patient	*M*	3.97	4.46	< .001	.95
	*SD*	.438	.571		
**Outcomes for medical professionalism**					
Professional attributes	*M*	4.17	4.14	.952	-.07
	*SD*	.450	.416		
Professional working	*M*	3.85	4.08	.100	.52
	*SD*	.472	.418		
The doctor as expert	*M*	3.71	3.85	.169	.27
	*SD*	.535	.495		
The global doctor	M	4.01	4.20	.339	.31
	*SD*	.596	.645		

Independent sample t-tests were computed to examine the effects of the exchange program on the Level 1 learning outcomes. The t-tests revealed significant effects of participation in the exchange program in the area of doctor-patient relationships, t(47) = 3..96, p < .001, d = 1.09, two-tailed, and effective communication in medical contexts, t(47) = 2.20, p = .033, d = 0.60, two-tailed. All other differences were not significant (p > = .10).

### RQ 3: which learning processes mediate the effects of exchange programs with regard to the self-assessment of medical skills?

The data from the *qualitative study* were combined with those from the *quasi-experimental study* to examine which processes mediated the effects of the exchange program on the self-assessment of medical skills.

In the *qualitative study*, the five interviewees made several statements about the way they learned, and it was clear that the social interactions and mentality of the local people as teachers were crucial for the learning process. These differences—an important learning criterion—stemmed from the cultural background of the local people and thus the perceived differences between the two cultures and the different learning environments. This assumption is confirmed by the statement of A “*I don’t think it makes much sense to send them [the students] somewhere, where everything is similar to how it is at home, but it makes sense to send them somewhere where it is different from home, that shapes you and you get a lot of benefit out of it*” (A) #00:23:32-3# (authors’ translation). In this area, we found a significant change in attitude in all interviewees and thus consider it to be a central effect of the exchange in regard to the process of learning. The perception of differences between the cultures resulted in a rethinking of the participants’ own views, attitudes, and actions. These differences were perceived primarily by interactions with local people. Such interactions were, as already pointed out repeatedly, sociocultural learning processes.

It is the “*food for thought*” (D) #00:13:03-9#, the “*becoming aware of it*” (C) #00:44:19-8#, the reflection or “*mirroring*” (B) #00:26:25-5# (authors’ translation) of the participants’ own thinking and behavior patterns that may have a large impact in many areas. The doctor-patient relationship is just one example. Some of the interviewees’ statements link back to what was said in the theoretical remarks about experienced crises. One example is the statement by interviewee C “*Overall, the whole experience was just very different. There, one often has moments of crisis in the hospital. Sometimes everything there was just completely different.*” (C) #00:12:45-0# (authors’ translation). To summarize, the perceived differences between the two cultures and the learning environments and the crisis experienced by participants seem to be important intermediaries of learning.

In the *quasi-experimental study*, we posed three questions about the learning process (to explore whether these processes can be identified as sociocultural learning). Twenty-four participants strongly agreed and 4 participants agreed with the statements “*By interacting with locals, I learned something that I would not have learned otherwise.*” (M = 4.72; SD = 0.80). In the item “*Through contact with Ethiopians, I benefited on a professional level*” (M = 4.10; SD = 1.08); 23 participants strongly agreed or agreed with and 5 participants partly agreed. None of the participants responded that statement three, “*The Ethiopians I had contact with knew more than I did in many areas,*” did not apply to them (M = 3.72; SD = 1.07). The mean values show that, for all items, participants’ responses were between “agree” and “strongly agree.”

Overall, the results indicate that most participants in the exchange program showed sociocultural learning during the exchange.

## Discussion and conclusion

The current study allowed the effects of exchange programs to be given a theoretical grounding by considering them in light of Vygotsky’s sociocultural learning theory. The process of interacting with local people raises awareness of the difference between one’s attitudes and opinions and those of the local people, i.e. learning is triggered by differences. Participants were confronted with challenges and situations of crisis, which resulted in a learning process [14,15]. This is particularly evident in regard to the doctor-patient relationship. The associated “crisis” was the participants’ questioning of their own position by reflecting on that of the Ethiopian people. Therefore, this process can be characterized as mirroring. There is a change in perspective, which plays an important role in successful learning, especially in sociocultural contexts.

Through the experience of a different kind of doctor-patient relationship in Ethiopian hospitals, German participants gained an insight into the importance of this area for the medical practice. Thus, the exchange program participants benefited in areas of competence that appear to be particularly important for clinical practice [17]. These areas have been focused on increasingly since the introduction of the new Medical Licensure Act in Germany in 2002 [19].

Both areas that showed significant outcomes—the doctor-patient relationship and the professional interaction with other doctors—are characterized by interaction. The time spent in Ethiopia resulted in a higher self-assessment of Tuning competencies concerning the ability to interact with others. The close connection between the learning process itself and what is being learned is noteworthy because it shows that the participants improved their interaction through interacting, i.e. through sociocultural learning.

On the basis of the studies by Mc Allister, et al. [2] and Stahl et al. [20], we assumed that participants in exchange programs increase their social and intercultural competencies in particular, with are extremely important competencies for their future practice in the healthcare system [21]. The results of the research presented here, however, show a significant gain in two of the four subject-specific Level 1 learning outcomes. In these two parts, gains were identified in subject-specific competencies. Linking these two findings, we can conclude that these subject specific competencies are closely linked with social competencies, particularly in the areas of doctor-patient relationships and subject-specific communication, and that the proportion of soft skills in these competencies is very high in comparison to the other subject-specific skills.

Fitzgerald [22] and Mc Allister et al. [2] demanded that medical exchange programs should provide not only general competencies detached from medical work, but also should promote subject-specific competencies, even if they have a high proportion of general competence areas. Our results support this demand by showing that the participants assessed their competencies on a higher level especially in fields with a combination of technical skills and social skills.

Balandin, Lincoln, Sen, Wilkins and Trembath [1] demanded that exchange programs should promote in particular those outcomes that are highly relevant in the participants’ occupational field. Again, this demand is supported by the study results. As the Tuning outcomes were designed for medical education in the EU [17], the results of the current study may only be applicable to the German health system; in other cultures, different competencies may be more important.

This survey gave a detailed insight into the effect of exchange programs by combining two different assessment methods and three research questions. This approach allowed us to explore the learning mechanisms through which exchange students gain certain specialist competencies. We found that the learning process is initiated by the perception of differences between the students’ own attitudes and opinions and those experienced in the foreign environment.The findings of this study should promote future studies on international exchange programs. We recommend that future studies combine qualitative and quantitative methods and consider the theory-based analysis of learning experiences.

### Limitations of the study

This study has some limitations because of the method of self-assessment. Participants in the exchange program may have assessed their competencies higher then did the control group, because they might be more self-positive, which may limit the interpretation of the data. One method of increasing the willingness to give truthful self-assessments is to guarantee full anonymity [23]. We applied this approach and informed study participants about the anonymity of the data. A meta-analysis by Falchikov and Boud (1989) found that self-assessments by students in advanced courses (like the students in the exchange program) have rather high reliability (i.e. correlation of r = .69 with an objective skill/knowledge test) [24].

Another limitation could be the rather low reliability of some of the scales. The reliability coefficients of some of the variables was sub-optimal (3 of the variables had an alpha < 0.7; see Table 1), which may have led to an underestimation of the effects (due to larger error variances).

The design of the quasi-experimental study does not guarantee that the gained competencies were attributable only to the exchange program, i.e. the experimental group may have gained competencies since the exchange. Nevertheless, the aim was to focus also on long-term effects. The time between participating in or applying for the exchange and the survey was the same for both the experimental and control groups, so that all participants had the same time to gain competencies.

### Ethical approval

Our research involved only questionnaires and de-identified interviews. All subjects participated on a voluntary basis and gave their consent for the results to be published. Therefore, ethical approval was not requested from our ethical review board. We give detailed information about the implementation of the study in the manuscript.

## Competing interests

The authors declare that they have no competing interests. The authors alone are responsible for the content and writing of the article.

## Authors’ contributions

All authors have made significant contributions to the article. FJ and KS coordinated the project from an educational perspective. MS attended the project as a medical education expert. FJ and MS are greatly involved in the exchange project with African universities. FJ designed the study. FJ, KS, and MS contributed to the interpretation of the results and the content of this paper and approved the final version. FJ collected and analyzed the data and wrote the first draft of the paper. All authors read and approved the final manuscript.

## Authors’ information

All authors work at the LMU Munich, Germany. FJ is an educational scientist at the Center for International Health CIH^LMU^, Department of Surgery; KS is an assistant professor at the Department of Psychology; and MS is a surgeon and medical educator at the Department of Surgery.

## Pre-publication history

The pre-publication history for this paper can be accessed here:

http://www.biomedcentral.com/1472-6920/14/43/prepub

## Supplementary Material

Additional file 1 Interview guideline for the survey. Click here for file

Additional file 2 Evaluation of reported gains in the level 1 Learning Outcomes. Click here for file

Additional file 3 Online questionnaire. Click here for file

## Previous presentations

“Effects of international exchange programs on subject specific competences.” Presentation: International Conference TL2011, Nigeria 06.09.2011.

“Promoting Medical Competencies through International Exchange Programs: What can medical students gain from an exchange program?” Presentation: International Conference IEASA 2012, Cape Town, South Africa 30.08.2012.
