# Supplementary material for: Promoting medical competencies through international exchange programs: benefits on communication and effective doctor-patient relationships
Source: BMC Med Educ. 2014 Mar 4;14:43. doi: 10.1186/1472-6920-14-43 (PMC3945959; doi:10.1186/1472-6920-14-43)
Supplement: Additional file 1 — Interview guideline for the survey. [file 1472-6920-14-43-S1.docx]

Hello, Thank you for taking the time for this interview. My name is Fabian Jacobs. I am studying Educational Science at LMU. Currently, I am writing my final thesis on the exchange program between LMU and Jimma University in Ethiopia in which you participated.
First, I would like to tell you about the goals of my work:
The aim of my thesis is to find out how the exchange program influenced the participants, i.e. you, personally or professionally.

Now, I want to give you some information regarding the interview:
The conversation will take about 60 minutes.

First, I will ask some general questions and then questions about your stay in Ethiopia. I am interested in your personal opinions, experiences, and also what kind of people you met. I am also very interested in the time since you returned from Ethiopia

If it is okay with you, I will audiotape the interview. All information will be depersonalized.

Do you have any questions so far?

I would appreciate it if you would take a moment to put yourself back into the time of the exchange.

I will start with some general questions:

1. How old are you?
2. What is your current occupation?
3. What year did you participate in the exchange program?
4. How long did you stay in Ethiopia?
5. Which languages do you speak?
6. To what extent had you already gathered longer-term international experience before your stay in Ethiopia?
   1. When?
   2. Where?
   3. How long?
   4. What did you do there?

Now, I would like to ask you some questions about your expectations of your stay abroad.

1. If you think back, what was your impression of Ethiopia before you went there?
2. What were your expectations for your stay abroad?
   1. Personally
   2. Professionally
3. Why did you apply for the exchange program?
4. What were your objectives in regard to your stay in Ethiopia?
   1. Personally
   2. Professionally

With regard to the time before the exchange, there is still something I would be interested in:

1. How much contact did you have with Ethiopians before the actual trip?

Now, I would like to ask some questions regarding your actual stay in Ethiopia:

1. What did you do there?
2. What did your daily routine look like?
3. Did you have far-reaching experiences in Ethiopia?
   1. Personally
   2. Professionally
4. What problems or challenges did you have to face?
5. What impressed you most during your stay?

I’m sure you met other people in Ethiopia and made new contacts. The following questions will help me to find out something about your social network.

1. Who were your most important contacts in Ethiopia?
2. How much contact did you have with the other German participants?
3. How much contact did you have with local people?
   1. Private
   2. Professional
4. In regard to the professional contacts, I would be interested to know the extent to which knowledge or expertise was exchanged?

Let us turn to the time **after** your return.

1. To what extent did you share your knowledge after your return from Ethiopia?
   1. With whom
   2. How
2. What specific abilities did you gain by staying in Ethiopia?

**The Tuning Level 1 Learning Outcomes**

Through surveys in the EU, a group of experts have determined various areas that are relevant for doctors in training.
Therefore, I would like to ask you to assess whether you gained something in the following areas through your stay in Ethiopia. I will ask these questions in English, because the international experts formulated them in English.

Did you gain anything in the following fields:

“Carry out a consultation with a patient”?

“Assess clinical presentations, order investigations, make differential diagnoses, and negotiate a management plan”?

“Provide immediate care of medical emergencies, including First Aid and resuscitation”?

“Prescribe drugs”?

“Carry out practical procedures”?

“Communicate effectively in a medical context”?

“Apply ethical and legal principles in medical practice”?

“Assess psychological and social aspects of a patient's illness”?

“Apply the principles, skills and knowledge of evidence-based medicine”?

“Use information and information technology effectively in a medical context”?

“Apply scientific principles, method and knowledge to medical practice and research”?

“Work effectively in a health care system and engage with population health issues”?

1. At the beginning of the interview you said that you had contact with Ethiopians before the actual trip to Ethiopia. I would be interested to know what impact this experience had on you?
2. Can you tell me some actual things that changed through this contact?
3. Now I would like to know in which areas your opinions have changed through the exchange?
4. And to what extent have your attitudes to certain things changed?
5. To what extent has your behavior been changed by your stay in Ethiopia?
   1. In your private life
   2. In your professional life
6. How much contact do you still have with people you met through the exchange?
   1. Germans
   2. Ethiopians
7. To what extent has the stay abroad influenced the choice of your current environment?
8. To what extent did the exchange change your approach to problems, situations and your work?
9. Finally, are there any other points that you feel are important to mention?

Thank you very much for the interesting interview.
