# Supplementary material for: Promoting medical competencies through international exchange programs: benefits on communication and effective doctor-patient relationships
Source: BMC Med Educ. 2014 Mar 4;14:43. doi: 10.1186/1472-6920-14-43 (PMC3945959; doi:10.1186/1472-6920-14-43)
Supplement: Additional file 2 — Evaluation of reported gains in the level 1 Learning Outcomes. [file 1472-6920-14-43-S2.docx]

Appendix B: Evaluation of the responses to questions in which participants reported gains in the Level 1 Learning Outcomes

| Tuning Level 1 Outcomes | Person A | Person B | Person C | Person D | Person E | Overall |
| --- | --- | --- | --- | --- | --- | --- |
| Carry out a consultation with a patient | Physicians do not talk to their patients | A lot of contact with the patient, diagnoses I have never seen before, but no significant gain | Had difficulties, because of lack of patient contact | Only partly, rarely questions to the patient | Did not learn much, because of language barriers. Altogether, there is no patient consultation in Ethiopia | If, then only minor increase - Language barrier - Patient contact plays no role |
| Assess clinical presentation, order investigation, make differential diagnoses, and negotiate a management plan | No gain | Differential diagnosis a little, but not yet benefited. In the other cases, no gain. | No gain | No gain | Germany: In training, you have little personal responsibility. Ethiopia: You get to deal directly with the patient (you have to perform all the necessary steps); negative: a lot of responsibility although you have little experience. | Up to E, no gains For E important point. The largest gains. |
| Provide immediate care of medical emergencies, including First Aid and resuscitation | No gain | No gain | No gain | No gain | One can learn a lot, also because you are not allowed to do it in Germany. | Except E, no gains |
| Prescribe drugs | No gain | No gain | No gain | No gain | Only medium gain with regards to the German system | Except E, no gains |
| Carry out practical procedures | No gain | No gain | No gain | No gain | More than in Germany, because one is really needed. Not like in Germany, where one rather just gets in the way. | Except E, no gains  E: Large gain in comparison to Germany. |
| Communicate effectively in a medical context | Gain, because one has to speak English | Much discussion on both sides about specific systems and syndromes and the way things are in Ethiopia. | Communication in general: yes; medically: a little. | Small gain | Little communication with patients; with colleagues, yes, especially in emergencies | The most gains |
| Apply ethical and legal principles in medical practice | No gain | No gain | Gains in learning to speak on an equal footing and in dealing with patients | No gain | They are just not interested in ethical principles, but one learns to appreciate that it’s different in Germany. | Small gains |
| Assess psychological and social aspects of a patient's illness | Just a little; patients from another culture. | High gain: syndromes have a different significance, and are dealt with differently and explained to the patient differently | In the villages, a little learned by seeing the living conditions and hygienic conditions | No gain | These are not taken seriously; only a little regarding the social environment | Three had gains; E had only small gains |
| Apply the principles, skills and knowledge of evidence-based medicine | No gain | No gain | No gain | No gain | Tried to apply them, but it was difficult and not really possible there. | No gains |
| Use information and information technology effectively in a medical context | No gain | Learned somewhat from the German support, but not extreme gains. | Seen how difficult it is to get information | No gain in the medical context | Little, because information is only sparse | No gains |
| Apply scientific principles, method and knowledge to medical practice and research | No gain | No gain | No gain | No gain | Research does not exist, no gain | No gain |
| Work effectively in a health care system and engage with population health issues | No gain | Small gain | No gain | No gain | Working independently, to explore disease areas that are not important for Germany (areas such as tropical medicine) | Small gain |
