# Supplementary material for: Promoting medical competencies through international exchange programs: benefits on communication and effective doctor-patient relationships
Source: BMC Med Educ. 2014 Mar 4;14:43. doi: 10.1186/1472-6920-14-43 (PMC3945959; doi:10.1186/1472-6920-14-43)
Supplement: Additional file 3 — Online questionnaire. [file 1472-6920-14-43-S3.docx]

Dear Participant,

Thank you for taking the time to complete this online questionnaire. This survey is part of a thesis that aims to investigate the influence of the exchange between LMU and Jimma University in Ethiopia. You will need about 10 minutes to answer all the questions. This questionnaire is completely anonymous

First, we would like to know some general things about you.

How old are you: ____

Sex:  male  female

Have you already completed your medical degree?:  yes  no

If not, which semester are you currently in?: ____

Did you participate in the exchange between Jimma University

and LMU?  yes  no

In which year did you participate in the exchange program?: ____

How long did you stay at Jimma University?: ____

*Sociocultural learning*

**To what extent do the following statements apply to your stay in Ethiopia?**

| 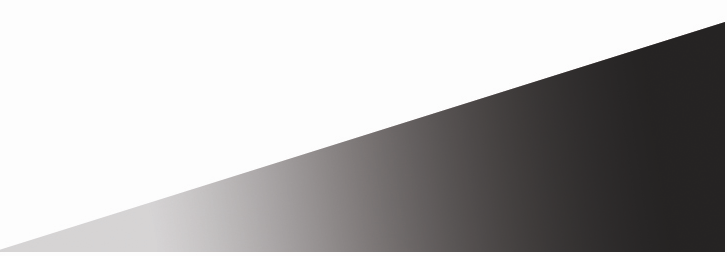 | | | | | |
| --- | --- | --- | --- | --- | --- |
|  | **Strongly**  **disagree** | **2** | **3** | **4** | **strongly**  **agree** |
| Through interacting with local people, I learned something that I otherwise would not have learned. |  |  |  |  |  |
| I benefited professionally through the contact with Ethiopians |  |  |  |  |  |
| The Ethiopians I had contact with knew more than I did in many areas. |  |  |  |  |  |

**The following questions focus on possible international experience that you may have had beyond private vacations. Have you been abroad (in addition to the visit to Ethiopia) as part of your medical training?**

yes  no

Which countries have you stayed in?

_____________________________________

_____________________________________

How long have you been abroad altogether?

_____________________________________

What did you do during your stay abroad?

_____________________________________

_____________________________________

**In the following questions, we ask you to assess your competencies.**

**Here you should specify to what extent the statements apply to you.**

*COMMUNICATION*

**To what extent do the following statements apply to you?**

| 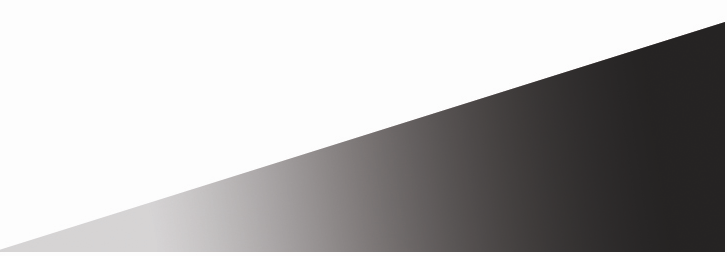 | | | | | | |
| --- | --- | --- | --- | --- | --- | --- |
|  | **Strongly**  **disagree** | **2** | **3** | **4** | | **strongly**  **agree** |
| I can talk appropriately with patients from other cultures |  |  |  |  |  | |
| I can communicate purposefully in a medical context |  |  |  |  |  | |
| I have had a lot of exchanges with other professionals about best practices in medical practice. |  |  |  |  |  | |
| My ability to communicate with colleagues is excellent |  |  |  |  |  | |
| I can have a good conversation with someone who needs an interpreter |  |  |  |  |  | |

*ETHICALLEGAL*

**To what extent do the following statements apply to you?**

| 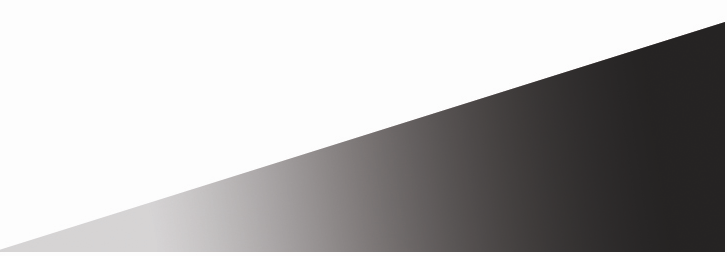 | | | | | |
| --- | --- | --- | --- | --- | --- |
|  | **Strongly**  **disagree** | **2** | **3** | **4** | **strongly**  **agree** |
| I find it easy to converse with patients on an equal footing |  |  |  |  |  |
| I know why it is important to follow ethical principles |  |  |  |  |  |
| I appreciate the German health care system |  |  |  |  |  |
| I appreciate the German guidelines for medical care |  |  |  |  |  |
| Ethical principles are essential for medical practice |  |  |  |  |  |
| I've thought a lot about ethical principles in medicine |  |  |  |  |  |

*PSYCHOLOGICALSOCIAL*

**To what extent do the following statements apply to you?**

| 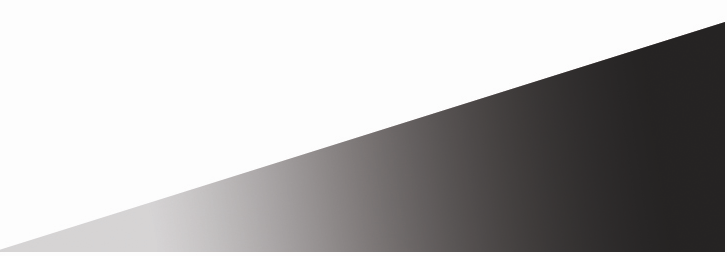 | | | | | |
| --- | --- | --- | --- | --- | --- |
|  | **Strongly**  **disagree** | **2** | **3** | **4** | **strongly**  **agree** |
| I can assess psychological aspects of a disease very well |  |  |  |  |  |
| I am able to understand and analyze social aspects of an illness very well |  |  |  |  |  |
| I know the consequences that diseases can have |  |  |  |  |  |
| Social factors are very relevant for diseases |  |  |  |  |  |
| Psychological aspects have a major impact on disease |  |  |  |  |  |

*CONSULTATION*

**To what extent do the following statements apply to you?**

| 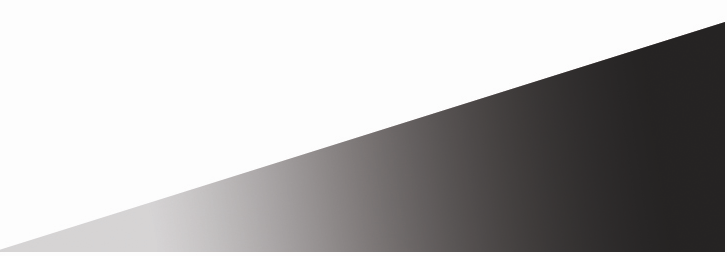 | | | | | |
| --- | --- | --- | --- | --- | --- |
|  | **Strongly**  **disagree** | **2** | **3** | **4** | **strongly**  **agree** |
| Patients from other cultures have a different understanding of the doctor-patient relationship |  |  |  |  |  |
| I know why the doctor-patient relationship is extremely important |  |  |  |  |  |
| So far, I have had a lot of contact with patients |  |  |  |  |  |
| I can provide explanation and assistance to a patient |  |  |  |  |  |
| I have a deep understanding of patients from other cultures |  |  |  |  |  |

**Outcomes for Medical Professionalism**

The final assessment deals with general competencies that have no specific medical orientation.

**Again, we kindly ask you to rate your competencies.**

*Professional attributes*

| 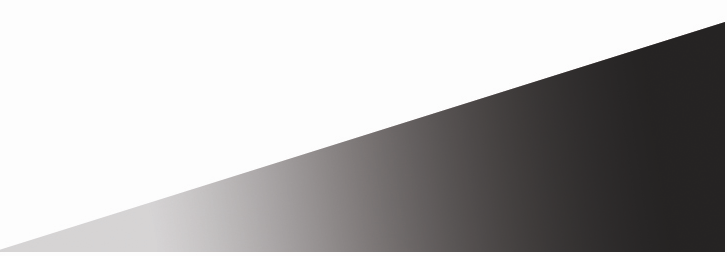 | | | | | |
| --- | --- | --- | --- | --- | --- |
|  | **Strongly**  **disagree** | **2** | **3** | **4** | **strongly**  **agree** |
| Probity, honesty, ethical commitment |  |  |  |  |  |
| Commitment to maintaining good practice, concern for quality |  |  |  |  |  |
| Critical and self-critical abilities, reflective practice |  |  |  |  |  |
| Empathy |  |  |  |  |  |
| Creativity |  |  |  |  |  |
| Initiative, will to succeed |  |  |  |  |  |
| Interpersonal skills |  |  |  |  |  |

*Professional working*

| 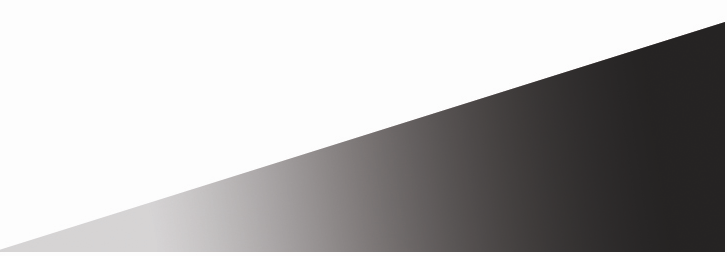 | | | | | |
| --- | --- | --- | --- | --- | --- |
|  | **Strongly**  **disagree** | **2** | **3** | **4** | **strongly**  **agree** |
| Ability to recognise limits and ask for help |  |  |  |  |  |
| Ability to work autonomously when necessary |  |  |  |  |  |
| Ability to solve problems |  |  |  |  |  |
| Ability to make decisions |  |  |  |  |  |
| Ability to work in a multidisciplinary team |  |  |  |  |  |
| Ability to communicate with experts in other disciplines |  |  |  |  |  |
| Ability to lead others |  |  |  |  |  |
| Capacity to adapt to new situations |  |  |  |  |  |
| Capacity for organisation and planning (including time management) |  |  |  |  |  |

*The doctor as expert*

| 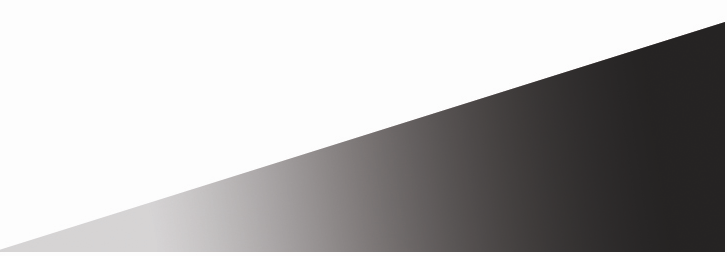 | | | | | |
| --- | --- | --- | --- | --- | --- |
|  | **Strongly**  **disagree** | **2** | **3** | **4** | **strongly**  **agree** |
| Capacity for analysis and synthesis |  |  |  |  |  |
| Capacity to learn (including lifelong, self-directed learning) |  |  |  |  |  |
| Capacity for applying knowledge in practice |  |  |  |  |  |
| Ability to teach others |  |  |  |  |  |
| Research skills |  |  |  |  |  |

*The global doctor*

| 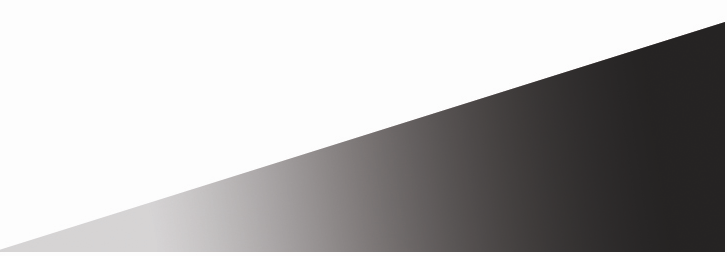 | | | | | | |
| --- | --- | --- | --- | --- | --- | --- |
|  | **Strongly**  **disagree** | **2** | **3** | **4** | | **strongly**  **agree** |
| Appreciation of diversity and multiculturality |  |  |  |  |  | |
| Understanding of cultures and customs of other countries |  |  |  |  |  | |
| Ability to work in an international context |  |  |  |  |  | |
| Knowledge of a second language |  |  |  |  |  | |
| General knowledge outside medicine |  |  |  |  |  | |

**Thank you very much**

**for you participation!**
